# The Role of Lumican in Ocular Disease

**DOI:** 10.1155/2013/632302

**Published:** 2013-07-24

**Authors:** Shahriar Amjadi, Kelly Mai, Peter McCluskey, Denis Wakefield

**Affiliations:** ^1^School of Medical Sciences, University of New South Wales, Sydney, NSW 2052, Australia; ^2^Save Sight Institute, University of Sydney, Sydney, NSW 2001, Australia

## Abstract

Lumican is keratan sulfate proteoglycan of the small leucine rich proteoglycan family. Through studies in animal models lumican has been found to be critical in maintaining corneal clarity. It maintains ordered collagen fibrils which are vital in keeping the cornea transparent. It may also be important in primary open angle glaucoma influencing aqueous outflow. Lumican deficiency in mice results in increased axial length with fibromodulin deficiency and thinner sclerae. There is evidence suggesting that this characteristic may be pertinent in humans and lumican gene polymorphisms could be related to high myopia. Lumican plays a fundamental role in inflammation and wound healing. It localises macrophages to the site of corneal injury and recruits neutrophils in lipopolysaccharide-induced keratitis in mice. It has also been shown to bind lipopolysaccharide which may be critical in inflammatory diseases such as uveitis. Lumican is also important in wound healing revealing decreased synthesis in scar tissue and mediating Fas-Fas ligand interactions. It is present in human placenta and amniotic membrane suggesting that it may ensure viable amniotic membrane grafts. Lumican may also be involved in the formation of posterior capsular opacification following cataract surgery. Research into the pivotal role of lumican in the pathogenesis of ocular disease has resulted in greater understanding of the key role which proteoglycans play in human disease.

## 1. Introduction

A normal ocular extracellular matrix is vital for ocular structure, homeostasis, and function. It is increasingly evident that it is also essential to maintain ocular immunologic status and immune privilege. A key component of the extracellular matrix is lumican, a proteoglycan that plays an important role in many structural, inflammatory, and disease processes. Animal studies in glaucoma, high myopia, inflammatory eye diseases, and wound healing indicate an important role for lumican in the pathogenesis of these common eye diseases. There are limited data from human studies.

In humans the gene for lumican (*lum*) is located on the short arm of chromosome 12q22. Lumican is an extracellular matrix protein of the small leucine rich proteoglycans (SLRPs). Within the SLRP family there are also decorin, fibromodulin, biglycan, and keratocan [1, 2]. Lumican, as with other SLRPs, has a molecular mass of approximately 40 kDa [3, 4] and is 338 amino acid residues in length [5, 6]. It consists of four domains, namely, (1) a signal peptide, (2) a negatively charged N-terminal domain, (3) tandem leucine-rich repeats, which contain the *N*-glycosylation sites for the keratan sulfate glycosaminoglycans (GAGs) and have a role in various molecular recognition processes [7], and a carboxyl terminal 50 amino acid residues in length with a disulfide-bonded cysteine residues 32 residues apart [4, 8].

Lumican was first characterised as a corneal keratan sulfate proteoglycan in chick cornea and was indeed named lumican in recognition of its role in corneal transparency [3]. Since then it has been characterised in other tissues including bovine aorta [9], bovine keratocytes [10], mouse cornea [11] as well as in human articular cartilage [5], kidney [12], and lung [13]. 

Lumican is critical for corneal clarity [14] by maintaining the strict collagen architecture of the cornea that is vital for corneal transparency [15]. Mice homozygous for lumican gene deletion have bilateral corneal opacity, along with skin laxity and fragility resembling an Ehlers-Danlos-type syndrome [16]. Transmission electron microscopy studies of these defective corneas revealed deregulated growth of collagen fibrils with a significant proportion of them being abnormally thick [16]. Further studies using confocal microscopy to quantify the opacity indicated a threefold increase in stromal back-scattering as well a 40% decrease in stromal thickness [14]. Transmission electron microscopy studies revealed collagen fibril abnormalities in the posterior stroma of lumican-deficient mice, while the anterior stroma was interestingly relatively unaltered [14], which may indicate a role for the posterior stroma in maintaining normal fibril architecture. This may be significant in understanding the formation of corneal opacities in certain conditions and after corneal surgery.

**Figure 1:**
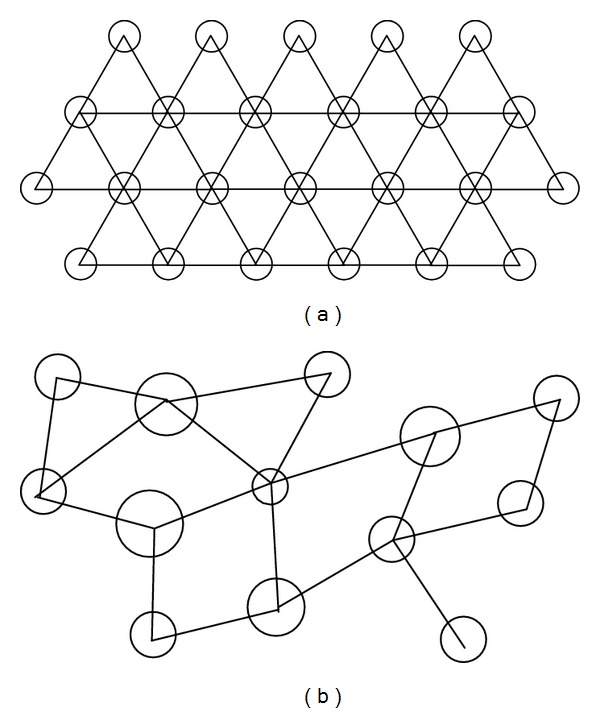
(a) Transverse view of corneal stroma with ordered arrangement of collagen fibrils (circles) with proteoglycan attachments anchoring the fibrils and maintaining their spatial order [21]. (b) Where there is a deficiency or defect in lumican, collagen fibrillar structure is not maintained.

Lumican-deficient mice show a significant disorder in the configuration and range of diameters of collagen fibrils compared to the regular packed fibrils of normal mice. This indicates disarray of stromal collagen architecture in corneas without lumican [17]. X-ray diffraction studies on keratocan-null mice revealed a similar disorderly structure [18], but mimecan-null mice displayed essentially normal corneal architecture [19]. Even in neonatal mouse corneas, deficiency of lumican led to less organised and thinner collagen fibrils [20]. Three-dimensional electron microscopic analysis of bovine cornea suggests that various intermolecular forces between collagen fibrils and proteoglycans, including lumican, are responsible for maintaining the normal fibrillar architecture and where there are deficiencies or defects in proteoglycans this would lead to a disruption in how the fibrils align themselves, leading to a disorganised array [21] (Figures 1(a) and 1(b)). These data from animal studies may indeed prove crucial in expanding our understanding of vision-threatening corneal disease characterised by corneal opacity. Thus far only one study looking at genes involved in central corneal thickness in humans did not find any single nucleotide polymorphisms (SNPs) associated with the lumican gene [22]. Clearly, however, more work needs to be done in this area as it may lead to breakthrough understandings of corneal disease and therapeutics.

## 2. Lumican and Glaucoma

Lumican has been implicated in regulating aqueous humor outflow. It has been shown that soluble extracellular proteoglycans contribute to aqueous humor outflow in the trabecular meshwork [23]. Comparison of the trabecular meshwork in normal eyes and eyes from patient with primary open angle glaucoma revealed that lumican was expressed over twice as much in the glaucomatous trabecular meshworks compared to normal eyes [24]. Even though the mechanisms by which lumican effects aqueous outflow have not been defined, it is reasonable to speculate that it is involved in maintaining, and possibly regulating, aqueous outflow resistance.

## 3. Lumican Structural Implications

Genetic studies of gene polymorphisms in man also support a role for lumican in myopia. Single nucleotide polymorphisms (SNPs) in the putative regulatory region of the *lum* gene have been associated with pathological myopia in man. Three SNPs were associated with pathological myopia in a study looking at Northern Han ethnic Chinese [25]. In a similar study looking at Taiwanese Han Chinese, two SNPs were observed to be significantly associated with high myopia, including one that was also present in the previous Northern Chinese study [26], as well as in other studies [27, 28], in which the polymorphism was rs3759223 (C→T). These results have not been confirmed in other studies looking at Southeastern Han Chinese [29], Northeastern Han Chinese [30], and Koreans [31]. These results indicate that the role of the lumican gene in the pathogenesis of myopia requires further study.

Lumican is a key component of the sclera and was initially found in murine sclera [32]. Lumican gene deletions affect the structural integrity of the sclera. For example, it was found that heterozygous and homozygous lumican-null mice displayed increased collagen fibril diameter compared to normal controls, and that homozygotes had increased diameter compared to heterozygotes [32]. These observations may have implications for human vision especially in relation to myopia. Given that increased axial length, retinal detachment and scleral thinning may be features of myopia, there is evidence that lumican may play a role in this disease. Utilising transgenic zebrafish, downregulation of the *zlum* gene in this species led to ocular enlargement resembling axial myopia [33]. This was attributed to the disruption of the collagen fibril arrangement, causing scleral thinning and weakness.

In human sclerae lumican has been found as a 70- to 80-kDa core protein that complexes covalently with aggrecan [34]. Interestingly, the amount of this lumican-containing high molecular weight complex was seen to increase in age, whereas the amount of uncomplexed lumican declined with age. This may indicate a role for lumican, as well as aggrecan, in age-related extracellular matrix changes [34] which may lead to myopia.

The potential role of lumican in myopia is also supported by animal studies. Mice homozygous null for both lumican and fibromodulin genes, for example, had a 10% increase in axial length, though no significant difference was observed for those mice homozygous null for just one of the genes [35]. The double null and both single null mutants had thinner sclerae compared to controls, which corresponded to fewer scleral lamellae in the all three strands of mutants seen by transmission electron microscopy. Double-null mice had frequent retinal detachment as well as the lumican-null mutants, though to a lesser extent [35]. 

## 4. The Immune System

Lumican plays a role in immunological function as well as regulating wound healing. Mouse peritoneal macrophages have been shown to attach and spread rapidly on the lumican core protein but only after removal of the keratan sulfate side chains [36]. It has previously been shown that diseased corneas, such as those with bacterial keratitis or keratopathy and healing stromal wounds, expression of highly sulfated keratan sulfate chains is reduced [37] or eliminated [38]. This decrease in keratan sulfation of lumican may serve to localise the macrophages to the diseased area on the cornea. Pharmacological agents that regulate macrophage localisation through their specific interaction with lumican could potentially lead to new treatments for limiting intraocular inflammation.

Studies have shown that lumican, as well as keratocan, play a role in lipopolysaccharide- (LPS-) induced innate immune response [39]. LPS is now recognised to be a pathogen associated molecular pattern (PAMP) that binds to Toll-like receptors on cells of the innate immune system and leads to an immune response and inflammation. Using a mouse model of LPS-induced keratitis, where mice injected with LPS show increased corneal thickness and haziness due to neutrophil invasion into the corneal stroma, it was shown that *Lum* ^−/−^ and *Kera* ^−/−^ mice displayed a significantly lower increase in corneal thickness compared to wild-type mice [39]. *Lum* ^−/−^ mice also developed increased corneal haze compared to *Kera* ^−/−^ and wild type. Reconstitution of expression of the keratin sulphate proteoglycans (KSPGs) in the cornea by use of viral vectors resulted in a normal neutrophil response to LPS, suggesting that the KSPGs' role in immune regulation is at the eye itself, rather than through changes in the neutrophils. Furthermore, these KSPGs were found to bind the chemokines, which are essential in the recruitment of cells to the site of inflammation. In another study it was shown that the CXCL1 chemokine bound to lumican, as well as keratocan, during corneal inflammation and that this may play a role in producing a chemokine gradient essential to migration of cells from the vascular system to the site of tissue damage [40]. These data suggest an important role for lumican in neutrophil recruitment in the pathogenesis of keratitis.

**Figure 2:**
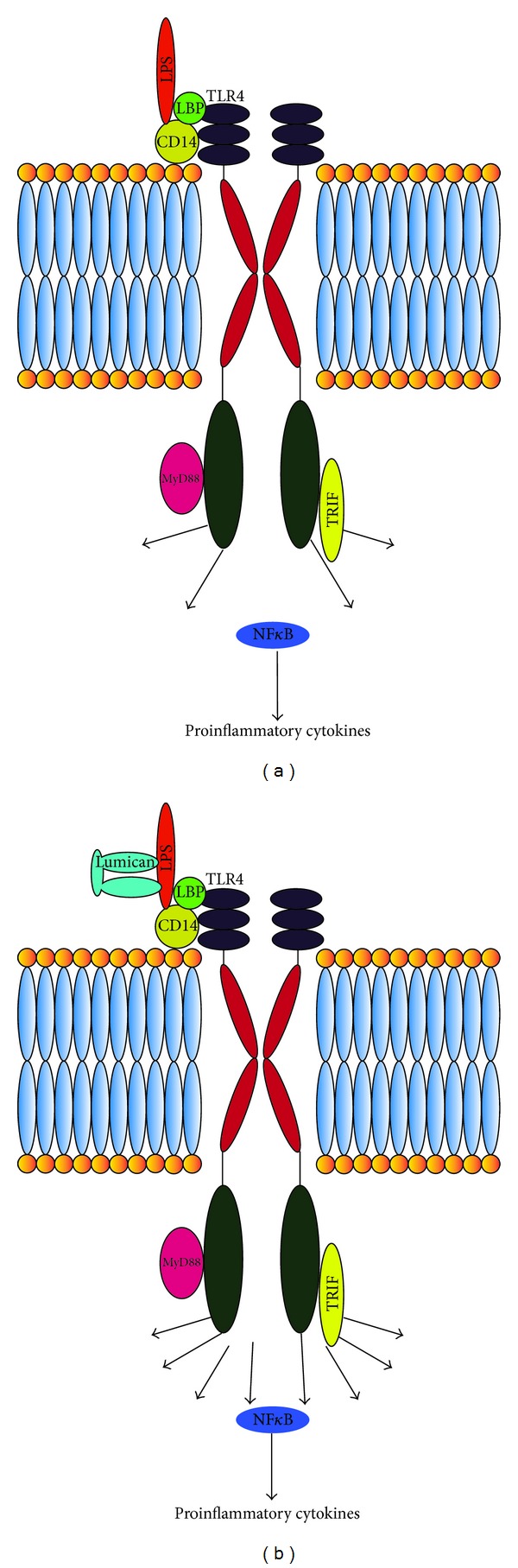
(a) When LPS binds to TLR4, with the cobinding molecule CD14, it leads to downstream production of proinflammatory cytokines via the NF-*κ*B transcription factor [53]. (b) Lumican by binding to LPS augments this response leading to increased production of proinflammatory cytokines [41].

Lumican's role in innate immunity also has been shown to involve the regulation Toll-like receptor 4 (TLR4) signaling [41]. TLRs recognise PAMPs, such as LPS, and TLR4 binds LPS, producing an inflammatory response. This is significant with respect to the eye as PAMPs have been implicated in inflammatory eye diseases such as uveitis [42]. Utilising primary cultures of lumican-deficient (*Lum* ^−/−^) macrophages, there was a decreased induction of the proinflammatory cytokines TNF*α* and IL-6, suggesting an impaired immune response. Furthermore, when compared to other PAMPs, macrophages did not show an impaired response in the presence of lumican deficiency indicating a specific role for lumican in the TLR4 pathway. Exogenous recombinant lumican partially salvages the decreased TNF*α* response. It was shown that lumican binds to LPS and TLR4 and coprecipitate with CD14, which is a cell surface protein that binds LPS and is involved in the presentation of LPS to TLR4 [41] (Figures 2(a) and 2(b)).

**Figure 3:**
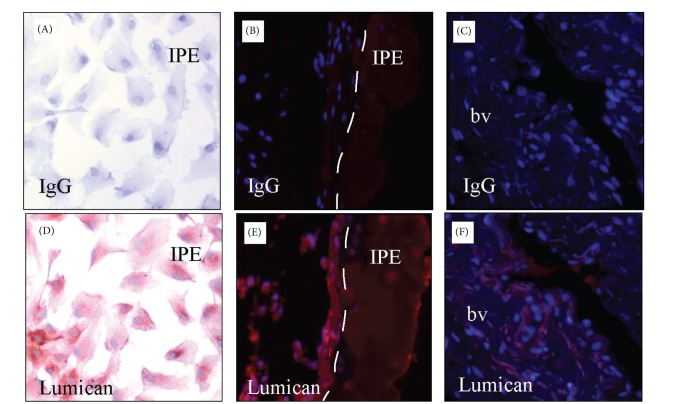
Lumican staining in human iris pigment epithelial cells (IPE) and iris. Human IPE cultures (A, D), iris tissues (B, E). Lumican staining (red) is present in IPE in vitro (D) and in vivo (E), in blood vessels (bv) (F).

Recent studies indicate that lumican can recruit polymorphonuclear neutrophils (PMNs) [43]. Comparing lumican null mice with wild types and novel transgenic mice expressing a lumican null/keratocan-lumican gene, more PMNs were observed in the stroma of injured corneas of the novel transgenics compared to the lumican null mice. It was also shown that wild type and keratocan-lumican transgenic mice display PMNs at the limbal region after central corneal injury, whereas there were none or very few in lumican null mice. There were no PMNs observed in the central cornea 60 minutes after the inciting injury to the cornea, which may indicate that though lumican may play a role in initially recruiting PMNs to damaged corneas, further signals are required to allow the invasion of PMNs into the stroma of injured corneas [43]. Given PMN recruitment is crucial to the inflammatory response in keratitis [44], lumican could play an active role in pathogenesis of infectious and noninfectious keratitis. Furthermore, our group has shown that lumican is present in iris, suggesting that there may be a similar role for lumican in recruiting inflammatory cells and promoting TLR mediated immune responses in the anterior uvea. (unpublished data) (Figure 3).

## 5. Wound Healing

Wound healing and inflammation are closely related and lumican has been shown to be important in corneal wound healing. Lumican synthesis is decreased in corneal scar tissue compared to normally growing stroma in animal studies [45]. Lumican has also been shown to be transiently expressed in murine corneal epithelial cells and anti-lumican antibodies delay corneal wound healing [46]. In another study, it was shown that lumican and keratocan are downregulated during wound healing at six weeks after epithelial debridement but returned to higher levels at 12 weeks [47] indicating that lumican expression during corneal wound healing is dynamic which may be significant in understanding the pathogenesis of corneal ulcers and other keratopathies.

Interestingly, lumican is also present in human placenta and amniotic membrane, which partially explains the efficacy of amniotic membrane grafts in corneal and conjunctival transplantation. Lumican was found in amniotic membrane using immunohistochemistry, Western blot, and gel electrophoresis [48]. The addition of purified human amniotic membrane lumican to cultured medium promoted reepithelialisation and enhanced cell proliferation [48]. Furthermore the application of amniotic membrane derived lumican to injured mouse corneas led to increased epithelial wound healing, the effect being more pronounced in lumican null mice compared to wild types [48].

In examining corneal wound healing at the molecular level, it has been postulated that lumican is significant in mediating Fas-Fas ligand interactions and apoptosis [49, 50]. This property of lumican may be important in corneal embryogenesis and in maintaining immune privilege [51]. Mouse embryonic fibroblasts (MEFs) from *Lum* ^−/−^ mice displayed increased rates of proliferation and decreased apoptosis [50]. The same was shown in *Lum* ^−/−^ mice stromal keratocytes at the postnatal period (day 10) and decreased apoptosis was also shown during corneal stromal wound healing in adult mice. Additionally, the protein p21^WAF1/CIP1^, which is a universal inhibitor of cyclin-dependent kinases, and protein p53, which plays a role in tumor suppression and is an upstream regulator of p21, were decreased in the MEFs of *Lum* ^−/−^ mice with p53 also being downregulated in the corneas of *Lum* ^−/−^ mice. Furthermore, Fas was shown to be considerably decreased in the MEFs and corneas of *Lum* ^−/−^ mice, which may indicate a possible mechanism for the decreased apoptosis. Another study looking at *Lum* ^−/−^-injured corneas showed decreased apoptosis by lower caspase-3/7 activity [49]. Though decreased Fas was also found, the mRNA levels were similar to that found in wild types suggesting lumican regulation of Fas at the protein level. Additionally, it was shown that lumican binds to Fas ligand. Given these observations it is possible that lumican may play a role in anterior segment dysgenesis.

Lumican has been implicated in the formation of cataracts and its surgical complications. It has been shown to accumulate in posterior capsular opacification (PCO) and anterior subcapsular cataract (ASC) [52]. In postoperative specimens, lumican is expressed in lens epithelial cells (LECs). Additionally, lens epithelial cells in PCO obtained 14 days or later after cataract surgery, as well as from ASC, were found to express the myofibroblast marker *α*-smooth muscle actin, which is a marker for the epithelial-mesenchyme transition (EMT). This is a key process that LECs undertake during wound healing. Furthermore, in lumican double-null mice LECs were epithelial cell-like five to ten days after injury, whereas LECs from heterozygous lumican single-null were fibroblast-like [52]. This indicates that lumican plays a functional role in the differentiation of cells during injury-induced EMT. This may indicate a mechanism of PCO, which is a common cause of loss of vision after cataract surgery.

Given these multiple roles of lumican it is worthy to consider it as a target in the treatment of ophthalmological disease and in minimising postoperative complications of modern cataract surgery. Developing pharmacological agents that block lumican from binding to TLR receptors may be a more targeted approach to minimising ocular inflammation, such as in uveitis, compared to corticosteroids that are used more commonly. Blocking lumican might also reduce the risk of developing PCO in patients after cataract surgery. Supplementary synthetic lumican could possibly be administered to restore corneal clarity where there is corneal scarring or even to limit the progression of myopia in patients who may be at risk of high myopia. Further study of lumican may lead to novel therapies for multiple ophthalmological conditions and should be actively pursued.

## 6. Conclusion

Lumican is expressed in the cornea, sclera, iris, lens, and trabecular meshwork. Animal studies reveal a vital role in maintaining corneal clarity and there is evidence suggesting that it may be associated with high myopia in humans. It has multiple regulatory roles at the cellular level, including suppressing cell proliferation by facilitating Fas-Fas ligand mediated apoptosis. With respect to its immune function, available data suggest a role in recruiting cells from the innate immune system, namely, neutrophils and macrophages, and that it binds chemokines and LPS. Further study to understand the role of lumican is needed to determine its activity in ocular extracellular matrix regulation and eye disease.
